# Association between serum and urinary environmental metal levels and major depressive disorder: a study based on logistic regression and quantile regression

**DOI:** 10.3389/fpubh.2024.1450983

**Published:** 2024-08-29

**Authors:** Qixuan Sun, Haiyang Ding, Chenxuan Lu, Lailai Yan, Bing Cao

**Affiliations:** ^1^Key Laboratory of Cognition and Personality, Faculty of Psychology, Ministry of Education, Southwest University, Chongqing, China; ^2^College of Computer and Information Science, Southwest University, Chongqing, China; ^3^CAS Key Laboratory of Mental Health, Institute of Psychology, Chinese Academy of Sciences, Beijing, China; ^4^Department of Psychology, University of Chinese Academy of Sciences, Beijing, China; ^5^Department of Laboratorial Science and Technology, School of Public Health, Peking University, Beijing, China

**Keywords:** case-control experiment, environmental metal, major depressive disorder, serum, urinary

## Abstract

**Background:**

Major depressive disorder (MDD) is a prevalent mental disorder globally. Increasing evidence suggests that Environmental Metal (EM) play a crucial role in MDD. Therefore, this study investigated the roles of barium (Ba), cesium (Cs), nickel (Ni), manganese (Mn), lead (Pb), mercury (Hg), cadmium (Cd), and tin (Sn) in the etiology of MDD.

**Methods:**

The study included 72 MDD patients and 75 healthy controls (HCs) from the Second People’s Hospital of Zhumadian, China. Inductively coupled plasma mass spectrometer (ICP-MS) measured the metal levels in serum and urine samples from both groups.

**Results:**

Significant differences in serum and urine levels of EMs were observed between MDD patients and HCs. After adjusting for age, gender, and BMI, logistic regression and quantile regression models revealed significant associations between EMs and MDD. In serum samples, higher Sn levels (OR = 1.22, *p* = 0.044) increased MDD risk, whereas higher Cs levels (OR = 0.02, *p* < 0.001), Cd (OR = 0.06, *p =* 0.047), and Mn (OR = 0.54, *p* = 0.016) decreased MDD risk. In urine samples, higher Ba levels (OR = 0.94, *p* = 0.015), Ni (OR = 0.87, *p* = 0.0024), Sn (OR = 1.62, *p* < 0.001), and Mn (OR = 0.77, *p* = 0.037) were significantly associated with MDD. Sn significantly positively predicted HAMD-24 scores at the 0.50 and 0.75 quantiles (β = 0.96, *p* = 0.018; β = 1.25, *p* = 0.008) as did Pb (β = 5.15, *p* = 0.001; β = 4.19, *p* = 0.004). Ba positively predicted depressive symptoms across all quantiles (all *p* < 0.05). Hg positively predicted HAMD-24 scores at the 0.50 quantile (β = 9.20, *p* = 0.050).

**Conclusion:**

These findings underscore EMs’ importance in depression, aiding in targeted interventions for varying degrees of depression and necessitating future studies to clarify causality and mechanisms.

## 1 Introduction

Depression is a persistent negative emotional state that can lead to various physical, cognitive, and social changes, interfering with daily life (1). Major depressive disorder (MDD) is a more severe form of depression, with primary diagnostic criteria including persistent depressed mood and loss of interest or pleasure in daily activities, with at least one symptom lasting for more than two weeks (2). According to a 2023 survey by the World Health Organization, approximately 280 million people worldwide suffer from depression (3).

Despite the fact that the pathogenesis of depression has not been fully elucidated, numerous studies have shown that various factors, such as genetics and environment, play significant roles in its onset (4). Among these factors, environmental metals (EMs) are believed to be closely related to the occurrence and progression of depression (5, 6). EMs encompass neurotoxic elements such as barium (Ba), cesium (Cs), nickel (Ni), manganese (Mn), lead (Pb), mercury (Hg), cadmium (Cd), and tin (Sn). These metals are present in air, food, and drinking water, posing significant health risks by disrupting various biological processes, particularly in the nervous system. Exposure to these metals can significantly impact neurodevelopment, neurobehavior, and cognitive function (7–9). While high levels of exposure are typically harmful, in optimal doses, Ni has some beneficial effects on human health. Understanding the nuanced relationship between metal exposure and neurological health is crucial, particularly in the context of varying exposure levels and their potential implications for mental health conditions like depression (5).

In recent years, an increasing number of studies have focused on exploring the potential relationships between EMs and depression. Preliminary evidence suggests that anomalies in the levels of certain EM may be associated with an increased risk of depression. For instance, changes in the concentration of urinary EMs such as Ni have been linked to an elevated probability of developing depression (10, 11). Although the levels of these metal in the human body are extremely low, they play essential roles in various physiological processes, including regulating enzyme activity, synthesizing hormones, and participating in neurotransmitter metabolism (12). A study based on the NHANES found that Ba and Sn in urine were positively associated with the severity of depressive symptoms (4). This finding suggests a potential dose–response relationship between these specific EMs and MDD, warranting further investigation. Manganese plays a crucial role as a co-factor for various enzymes, participating in bone development, blood coagulation, and other processes (10). A study highlights Cs as one of the crucial metals identified in the multivariate analysis for classifying depression (13). While cadmium, mercury, and Pb are typically regarded as toxic substances, research has shown that they may exhibit regulatory effects on certain biological processes at extremely proper doses (14). Other studies have found that Cd and Pb may participate in the pathophysiological processes of depression by inducing oxidative stress, affecting neurotransmitter metabolism, and other mechanisms (15, 16). However, the specific roles of some EMs, such as Sn and Ba in the context of varying MDD severity levels remain largely unexplored. While the NHANES study (4) points to a positive association, it does not delve into the nuances of how Sn and Ba abnormalities might differ across mild, moderate, and severe MDD. Understanding these potential distinctions is crucial for developing targeted interventions and personalized treatment strategies. Therefore, the evidence is not yet conclusive, and further research is needed to elucidate the relationships between these EMs and depression.

While these studies provide valuable insights, research on the relationship between EM and MDD is still in its early stages. Most existing research employs regression models like logistic or linear regression, focusing primarily on average effects. This approach often misses how metal exposure affects individuals at different levels of depression. Our study advances this research by using quantile regression, which focuses more on the variations caused by changes in the independent variables at an individual level (17, 18). This method allows us to explore differences among individuals with varying degrees of depression more precisely, building on the average effects identified by logistic regression to provide a more detailed analysis (19).

Furthermore, research on the analysis of EMs in urine is still a significant blank spot. Most current studies concentrate on serum, hair, or nail samples, which cannot comprehensively reflect the status of EMs within the body (20, 21). Urine, as a non-invasive and easily collectible sample, can provide valuable insights into the metabolism, excretion, and potential relationships between EMs and depression symptom fluctuations (22, 23). This research gap highlights the need for comprehensive studies that include urine EMs analysis to better understand the role of EMs in the pathophysiology of depression.

Therefore, this study aims to conduct a case–control study, comparing the concentrations of various EMs (including Ba, Cs, Ni, Mn, Pb, Hg, Cd, Sn) in the urinary and serum of MDD patients and healthy controls (HCs). We will apply quantile regression models to further explore how different levels of EMs mediate the odds of MDD and the severity of symptoms.

Based on the research objectives, the study posits the following hypotheses:

*H1*: Significant differences in the concentrations of select EMs in urine and serum are observed between patients with MDD and HCs.

*H2*: We hypothesize that elevated serum levels of Pb, Hg, Ba, Sn will be positively associated with MDD, while higher urinary levels of Mn, Ni will be negatively associated with MDD, after adjusting for potential confounders. Furthermore, we hypothesize that the strength and direction of these associations will differ between serum and urine samples.

*H3*: The patterns of some EMs, such as Sn and Ba abnormalities vary across different severity levels of MDD. Specifically, varying levels of EMs exhibit differential associations with HAMD-24scores.

## 2 Methods and materials

### 2.1 Subjects

Between March 2022 and January 2023, MDD patients were recruited from the Second People’s Hospital of Zhumadian City, Henan Province, China. Volunteers without any mental conditions (referred to as HCs) were also recruited from the health examination population at the same hospital’s physical examination center. All participants were enlisted from the same area and time frame as the MDD group. Informed consent was obtained from all participants, which included 147 MDD cases and control group members. The study sample comprised 36 female and 36 male MDD patients.

### 2.2 Ethical approval

This study received approval from the Medical Ethics Committee of the Second People’s Hospital of Zhumadian City, Henan Province (Approval No. IRB-2021-006-02) and was conducted in accordance with the Declaration of Helsinki.

### 2.3 Collection of basic and clinical information

The data was collected by professionally trained scientific researchers under the guidance of clinical doctors in the hospital. Detailed demographic and clinical data were collected for all participants, including gender, age, body mass index, occupation, marital status, family psychiatric history, parents’ educational levels, and parents’ marital status, as well as basic information from infancy and childhood. The severity of psychiatric symptoms was assessed using the HAMD-24 scale. The HAMD-24 scale is a widely used clinician-administered depression assessment scale that has been shown to have a strong correlation with MDD severity. In conjunction with routine blood, biochemical, and urine tests were performed.

### 2.4 Inclusion and exclusion criteria

The criteria for including HC are as follows: (1) Comparable in gender, age, and residence to the MDD group, with no mental disorders per DSM criteria, and a HAMD-24 score under 20; (2) At least a primary school education; (3) Normal results in standard medical tests such as hematology, urinalysis, fecal analysis, liver function tests, fasting blood glucose, renal function tests, chest X-ray, and ECG.

The criteria for including individuals with Major Depressive Disorder (MDD) are as follows: (1) MDD diagnosis by trained psychiatrists based on DSM-V criteria, with the current depressive Episode confirmed by the Mini International Neuropsychiatric Interview (M.I.N.I. 5.0); (2) At least a primary school education; (3) Significant depression, indicated by a HAMD-24 score of 20 or higher; (4) Participants aged 18–60 years, without gender restrictions.

The exclusion criteria for both groups are identical: (1) History of organic brain diseases or diagnosed neurological disorders, such as Parkinson’s disease, cerebral hemorrhage, large cerebral infarction, encephalitis, or epilepsy; (2) Severe, clinically significant, or unstable medical conditions affecting the liver, kidneys, gastrointestinal system, respiratory system, cardiovascular system, endocrine system, blood, nervous system, genitourinary system, musculoskeletal system, or metabolism; (3) Intellectual disability; (4) History of alcohol, drug, chemical, substance, or psychoactive substance abuse; (5) Impaired vision or hearing; (6) Pregnant or lactating women.

### 2.5 Sample collection and analysis

Sample collection was performed in the morning following a 12-h fasting period. Approximately 8.5 mL of venous blood was drawn for serum separation, and around 10 mL of first-morning urine was collected. Serum samples were obtained by venipuncture using gold inert separation gel containers, centrifuged at 4°C to separate the supernatant, and stored at −80°C for EMs analysis. For the experimental procedure, 0.1 mL of serum (or urine) was placed into a 2 mL centrifuge tube. Then, 0.1 mL of an internal standard containing indium (In), rhenium (Re), and 1.8 mL 1% nitric acid was added. The mixture was vortexed and measured using Perkin-Elmer Sciex Elan DRC II ICP-MS and Agilent 7700 × ICP-MS for EMs (including Ba, Cs, Ni, Mn, Pb, Hg, Cd, and Sn) analysis.

### 2.6 Statistical analysis

Statistical analyses were performed using RStudio version 4.1.2. All tests were conducted with a 95% confidence interval (CI), and significance was determined with a two-tailed *p*-value of less than 0.05. Continuous variables were described using either the mean and standard deviation (SD) or the median and interquartile range (IQR), while categorical variables were presented as frequencies and percentages (N, %). A Chi-square test was employed to analyze the frequency distribution of categorical data between the two groups. Before conducting inferential statistics, we used the Shapiro–Wilk test to assess the normality of all continuous variables to ensure they followed a normal distribution. Given that the concentrations of all EMs did not follow a normal distribution, they were summarized using the median and the lower and upper quartiles. We used the Mann–Whitney U test to assess the differences between groups to compare non-normally distributed continuous variables. The analysis also accounted for covariates such as age, gender, and BMI.

In addition, logistic regression and quantile regression analyses were conducted. Logistic regression was used to examine the odds ratios (OR) of developing MDD. Quantile regression was applied to evaluate HAMD-24 scores at the 25th, 50th, 75th, and 95th percentiles, providing insight into how various factors influence different severity levels of depressive symptoms across the score distribution. This comprehensive approach allowed for a detailed understanding of the factors associated with MDD and its severity. Spearman’s correlations analysis was used to examine correlations between different EMs and scales’ scores.

## 3 Results

### 3.1 Demographic characteristic

In this study, a total of 72 patients with MDD and 75 healthy controls were included. The Chi-square test was used to assess the statistical significance of differences between the two groups. The mean age (standard deviation) of MDD patients and HCs was 39.30 ± 15.50 years and 41.90 ± 6.90 years, respectively. There were statistically significant differences between the two groups in HAMD-24 scores (MDD patients: 26.80 ± 6.00; HCs: 2.10 ± 1.50; *p* < 0.001) and body mass index (BMI) (cases: 22.20 ± 4.30 kg/m^2^; HCs: 23.90 ± 2.10 kg/m^2^; *p* = 0.004) (see Table 1). Age and gender distribution did not differ significantly among participants (both *p*-values > 0.05).

**Table 1:** Demographic characteristics of the subjects.

Variable	HCs (*n* = 75)	MDD (*n* = 72)	*p*-value
Age, mean ± SD	41.9 ± 6.90	39.3 ± 15.5	0.178
Sex (female/male); *n*/%	49/26 (65.3/34.7)	36/36 (50.0/50.0)	0.060
HAMD-24 total score, mean ± SD	2.10 ± 1.50	26.8 ± 6.00	<0.001
BMI, kg/m^2^, mean ± SD	23.9 ± 2.10	22.2 ± 4.30	0.004

### 3.2 Difference-in-difference test for EMs levels

**Figure 1:**
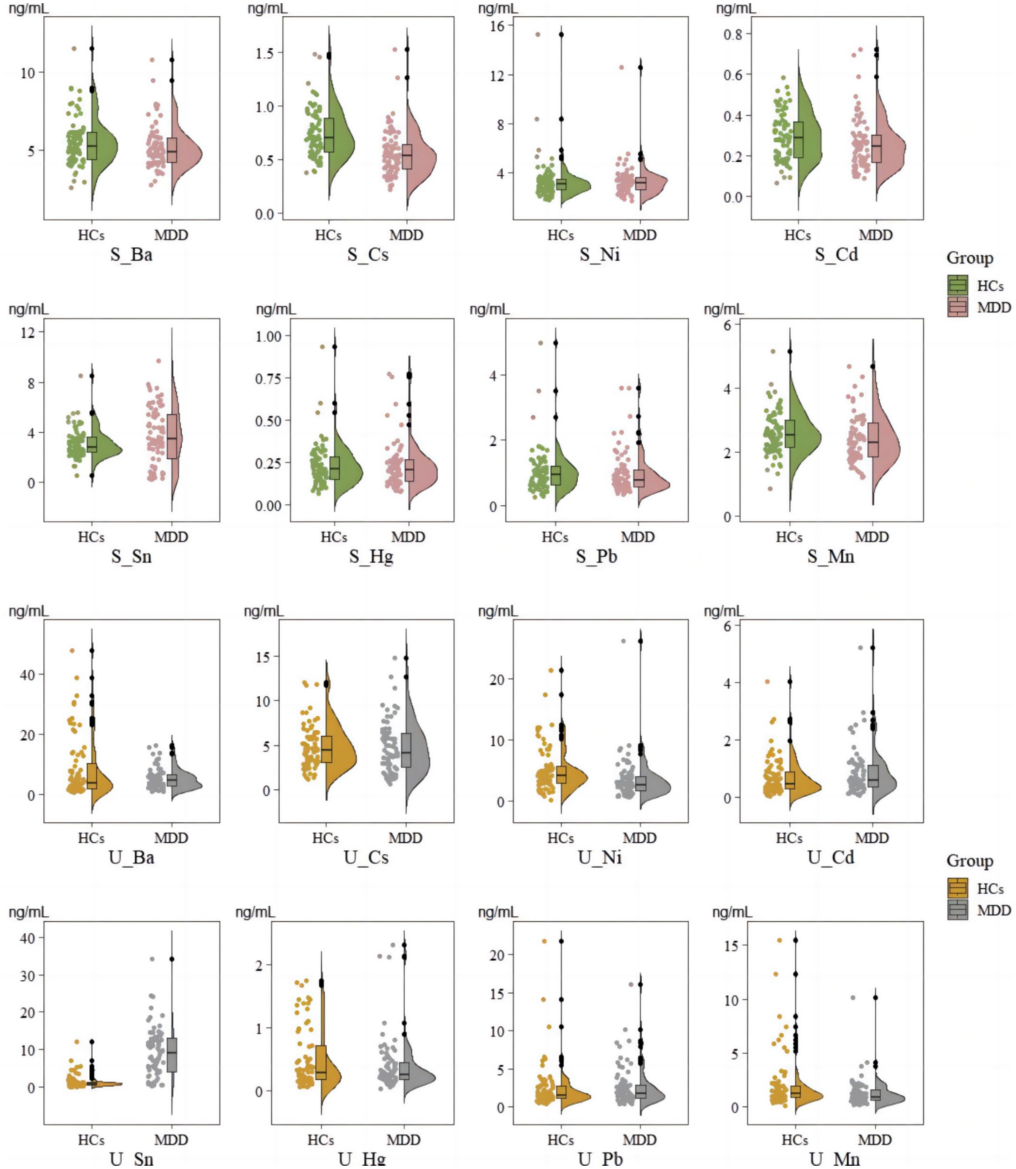
Comparison of levels in serum and urinary samples between MDD and HCs. S_means EM in serum; U_ means EM in urinary.

We used the Mann–Whitney U test to assess the differences between groups. This study found that, compared to HCs, patients with MDD exhibited significantly lower concentrations of Cs and Cd in serum samples, while Sn concentrations were significantly higher. In serum samples, the median concentration of Cs in the MDD group was 0.53 ng/mL (IQR: 0.40–0.65 ng/mL), compared to a median of 0.70 ng/mL in HCs (IQR: 0.57–0.90 ng/mL), *p* = 0.083. Similarly, Cd levels were lower in the MDD group, with a median of 0.25 ng/mL (IQR: 0.16–0.31 ng/mL), whereas the HCs had a median of 0.29 ng/mL (IQR: 0.19–0.37 ng/mL), *p* = 0.017. Conversely, the concentration of Sn was higher in the MDD group, with a median of 3.45 ng/mL (IQR: 1.82–5.41 ng/mL) compared to 2.77 ng/mL (IQR: 2.38–3.60 ng/mL) in the HCs, *p* = 0.026. There were no statistically significant differences in other EMs’ concentrations between the two groups (*p >* 0.050) (see Figure 1).

In urine samples, MDD patients showed significantly lower concentrations of Ni and significantly higher concentrations of Sn. Ni levels were significantly lower in MDD patients, with a median of 2.65 ng/mL (IQR: 1.63–4.05 ng/mL), compared to 4.08 ng/mL (IQR: 2.93–5.82 ng/mL) in the HCs, *p* = 0.006. Additionally, the concentration of Sn was significantly elevated in MDD patients, with a median of 9.07 ng/mL (IQR: 3.98–13.35 ng/mL) versus 0.89 ng/mL (IQR: 0.55–1.24 ng/mL) in HCs, *p* < 0.001. Other urinary elements, including Cs, Hg, Pb, and Mn, showed no significant differences between the two groups (*p >* 0.050) (see Figure 1).

### 3.3 Logistic regression model for EMs levels

**Figure 2:**
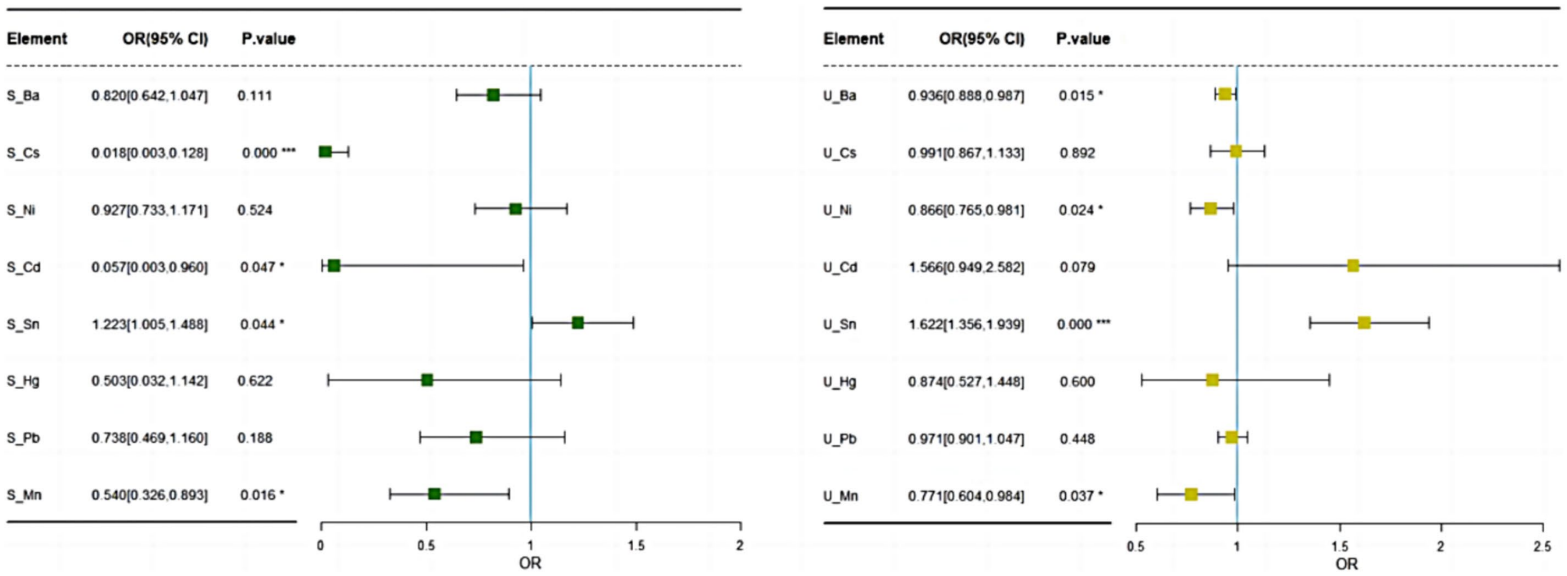
Logistic regression results of EMs in serum and urinary after adjusting for age, gender, and BMI.

After adjusting for age, gender, and body mass index (BMI), logistic regression analysis was conducted on serum and urine samples. The results indicate significant associations between certain EMs and the incidence of MDD. In serum samples, the elements Sn (OR = 1.22, 95% CI: 1.0–1.49, *p =* 0.044), Cs (OR = 0.02, 95% CI: 0.00–0.13, *p* < 0.001), Cd (OR = 0.06, 95% CI: 0.00–0.96, *p =* 0.047), and Mn (OR = 0.54, 95% CI: 0.33–0.90, *p =* 0.016) were significantly associated with MDD. Higher serum Sn concentrations were linked to an increased odds of MDD, indicating that exposure to Sn significantly raises the risk. Conversely, higher serum concentrations of Cs Cd, and Mn were associated with a lower odds of MDD, suggesting these elements may have protective effects. No significant associations were found between the levels of Pb, Ni, and Hg in serum and the incidence of MDD (see Figure 2).

In urine samples, the metals Ba (OR = 0.94, 95% CI: 0.89–0.99, *p* = 0.015), Ni (OR = 0.87, 95% CI: 0.37–1.07, *p =* 0.024), Sn (OR = 1.62, 95% CI: 1.36–1.94, *p <* 0.001), and Mn (OR = 0.77, 95% CI: 0.69–0.98, *p =* 0.037) were significantly associated with MDD. Higher urine levels of Ba, Ni, and Mn were found to be significantly associated with a lower odds of developing MDD, whereas elevated urine levels of Sn increased the odds of developing MDD, though its clinical significance might be limited. There were no significant associations between the levels of Pb, Cs, Hg, and Cd in urine and the incidence of MDD (see Figure 2).

### 3.4 Quantile regression model based on HAMD-24 scale score

**Figure 3:**
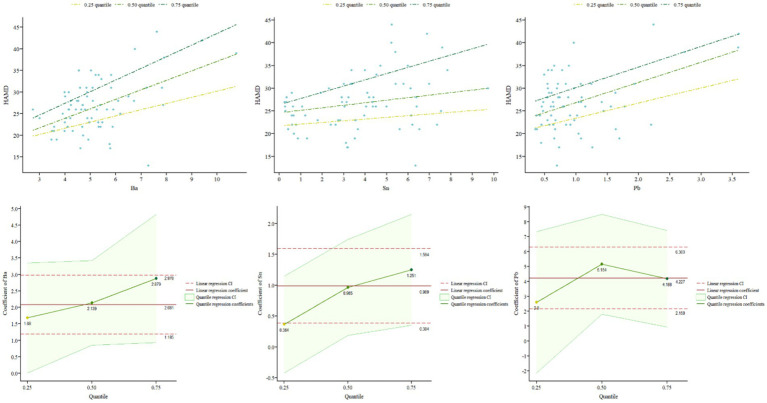
Quantile regression coefficients and curves in serum and urinary.

In order to further explore whether the influence of different elements varies with different severity levels of the disease, this study used a quantile regression model. After adjusting for covariates such as age, gender, and BMI, quantile regression models were established for serum and urine samples from MDD patients. Table 2 reports the regression results at different quantiles (0.25, 0.50, 0.75) of HAMD-24 scores. According to the quantile regression analysis results, in serum samples, the element Sn significantly positively predicted HAMD-24 scores at the 0.50 and 0.75 quantiles (β = 0.96, *p =* 0.018; β = 1.25, *p =* 0.008), and as the severity of depression increased, the regression coefficients of the model increased, indicating a higher impact of Sn on depressive symptoms with increasing severity. Pb also significantly positively predicted depressive symptoms at the 0.50 and 0.75 quantiles (β = 5.15, *p =* 0.001; β = 4.19, *p =* 0.004). The impact of Ba across all quantiles was significant (β = 1.68, *p* = 0.048; β = 2.14, *p =* 0.002; β = 2.88, *p =* 0.005), indicating that Ba can significantly positively predict HAMD-24 scores regardless of depression severity. Moreover, the influence of Ba on depression severity increases as the level of depression increases (see Figure 3). Hg significantly positively predicted HAMD-24 scores at the 0.50 quantile (β = 9.20, *p =* 0.050) but did not show significant effects at other quantiles (all *p >* 0.050). The effects of elements Cs, Ni, Cd, and Mn were not significant at any quantile (all *p >* 0.050), indicating that these elements do not significantly predict HAMD-24 scores.

**Table 2:** Quantile regression results in serum and urinary of the subjects.

TMEs	Quantile	Serum			Urinary		
		β	*t*	*p*	β	*t*	*p*
Ba	25	1.68	1.97	**0.048**	0.03	0.11	0.916
	50	2.14	3.26	**0.002**	−0.06	−0.28	0.782
	75	2.88	2.89	**0.005**	−0.30	−0.76	0.447
Cs	25	−3.98	−1.69	0.247	0.21	0.46	0.649
	50	−3.12	−0.91	0.366	−0.05	−0.14	0.888
	75	−2.04	−0.42	0.673	−0.02	−0.04	0.967
Ni	25	0.37	0.47	0.637	0.00	0.00	0.998
	50	0.34	0.53	0.598	−0.20	−0.44	0.659
	75	0.09	0.12	0.905	−0.11	−0.15	0.882
Cd	25	−2.44	−0.34	0.731	0.46	0.37	0.709
	50	−1.97	−0.29	0.775	0.37	0.31	0.758
	75	6.92	0.58	0.562	−0.10	−0.09	0.930
Sn	25	0.36	0.95	0.348	−0.02	−0.13	0.897
	50	0.96	2.42	**0.018**	−0.01	−0.09	0.927
	75	1.25	2.75	**0.008**	−0.08	−0.42	0.675
Hg	25	5.28	0.63	0.531	0.04	0.02	0.981
	50	9.20	2.00	**0.050**	−0.31	−0.25	0.807
	75	3.77	0.45	0.657	0.17	0.11	0.910
Pb	25	2.60	1.25	0.215	−0.32	−0.75	0.454
	50	5.15	3.36	**0.001**	−0.12	−0.34	0.736
	75	4.19	2.99	**0.004**	−0.26	−0.51	0.614
Mn	25	0.21	0.12	0.906	0.12	0.09	0.928
	50	2.73	1.57	0.120	0.46	0.41	0.681
	75	2.05	1.24	0.221	−0.05	−0.05	0.964

Bold values indicate statistical significance at the *p* < 0.05 level.

In addition, in urine samples, the effects of all contained metals were not significant at any quantile (all *p >* 0.050), indicating that these elements do not significantly predict HAMD-24 scores (see Table 2).

### 3.5 Correlation analysis

In our Spearman correlation analysis, we demonstrated significant relationships between EMs concentrations in serum and urine samples and various psychological scales’ scores. Specifically, Sn in both serum and urine showed a significant positive correlation with the scores of the Hamilton Depression Rating Scale-24 (HAMD-24) (*r* = 0.19), the Snaith-Hamilton Pleasure Scale (SHAPS) (*r* = 0.14), the General Anxiety Disorder-7 (GAD-7) scale (*r* = 0.18), and the Pittsburgh Sleep Quality Index (PSQI) (*r* = 0.19), suggesting that higher levels of Sn are associated with more severe symptoms of depression, anhedonia, anxiety, and sleep disturbances.

**Figure 4:**
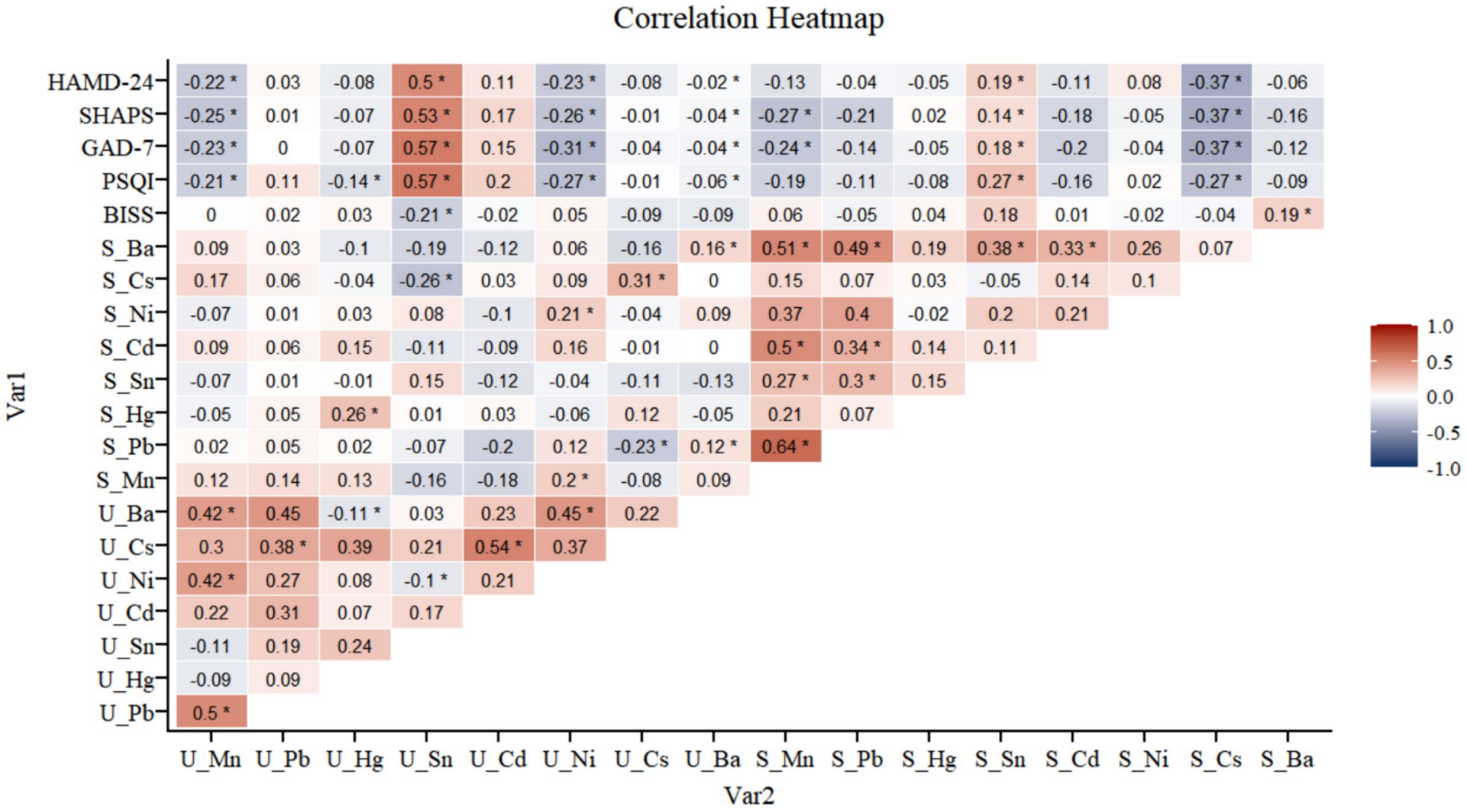
Spearman correlations of EMs levels in serum and urinary with MDD.

Additionally, we observed that Cs in serum exhibited a noteworthy inverse correlation with the HAMD-24 scores (*r* = −0.26), indicating a potential protective role against depression symptoms. This pattern was also seen with other metals in urine samples; Mn (*r* = −0.24), Ni (*r* = −0.21), and Ba (*r* = −0.25) all correlated inversely with HAMD-24, SHAPS, GAD-7, and PSQI scores. In urine, Ba and Mn exhibited a notably strong positive correlation (*r* = 0.45). In serum, Ni and Cd also showed a significant positive correlation (*r* = 0.34). Conversely, serum Cs and urine Ni were found to have a significant negative correlation (*r* = −0.26) (see Figure 4).

## 4 Discussion

### 4.1 Core findings of the study

In this study, our primary findings indicated significant differences in the concentrations of specific EMs between these two groups. Specifically, the concentrations of Cs and Cd were lower in the serum of MDD patients, whereas the concentrations of Sn were higher. In urine samples, Sn and Ba levels were significantly elevated, while Ni levels were significantly reduced in MDD patients. The results confirm our hypothesis H1. Additionally, our findings partially support the hypothesized H2. Higher serum concentrations of Sn were associated with an increased odds of developing MDD, while higher concentrations of Cs, Cd, and Mn were associated with reduced odds. Although we found higher cadmium Cd concentrations in the blood of subjects with MDD, Cd levels can be influenced by various factors, including states of anxiety, anemia, chronic obstructive pulmonary disease, zinc, iron, and calcium deficiencies, and ingestion of microplastics, and smoking (24–27). Urine evidence suggested that exposure to Ba, Ni, and Mn had a protective effect against MDD, whereas exposure to Sn increased the risk. This finding supports our hypothesis regarding the protective effect of urinary Mn and Ni against MDD. However, our results also diverged from the hypothesis in several ways. Contrary to our expectations, higher serum levels of Cs, Cd, and Mn were linked to reduced MDD odds, not increased odds as hypothesized for Pb, Hg, and Ba. Moreover, we anticipated a positive association between urinary Ba and MDD, but our findings indicated a protective effect.

Our findings provide compelling support for the hypothesis that patterns of certain EM abnormalities, specifically Sn and Ba, vary across different severity levels of MDD. Quantile regression analysis further indicated that serum Ba concentration significantly predicted HAMD-24 scores at any quantile, meaning that Ba levels significantly impact depression severity across all levels of depression severity. As the severity of depression increased, the impact of Ba on depression severity became more pronounced. Moreover, excessive levels of Hg, Sn, and Pb in the serum had significant negative effects on the occurrence and progression of varying degrees of depression.

### 4.2 Key elements with notable differences and potential mechanisms

Our findings indicated that serum Barium levels significantly positively predicted depression severity, and this association became stronger as depression severity increased. This observation aligns with a previous study (28) which found that high concentrations of barium were associated with an increased risk of depressive symptoms in older adult women, further supporting a potential link between barium levels and depression. Ba can accumulate in organisms through the food chain. High doses of Ba salts are toxic, causing gastrointestinal irritation, kidney damage, and arrhythmias (28). Ba toxicity is linked to its ability to disrupt potassium channels in cells, which can affect neuronal function and potentially lead to neurotoxicity. This neurotoxicity might exacerbate depressive symptoms by disrupting normal brain function (29). In urine, Ba and Mn exhibited a notably strong positive correlation, suggesting a possible common pathway or similar excretory mechanisms in the body.

Tin was found at higher concentrations in both serum and urine samples of individuals with MDD compared to healthy controls. Expose to the high Sn levels, especially from organic compounds, can cause various symptoms including headaches, dizziness, and mental confusion, which are closely linked to depression (30, 31). Sn’s neurotoxicity might involve the disruption of neurotransmitter systems and oxidative stress, which can impair cognitive and emotional regulation, thereby exacerbating depressive symptoms (30). These findings underscore the potential of Sn as a biomarker for MDD, suggesting that chronic exposure to Sn might lead to cumulative neurotoxic effects (19).

Nickel, an essential trace element involved in enzyme functions and neurotransmitter activity, was found at lower concentrations in MDD patients’ urine. Higher urinary Ni levels were significantly correlated with reduced odds of MDD, which reflect better systemic Ni regulation or reduced Ni retention in tissues, potentially affecting neurological health positively. In serum, Ni and Cd also showed a significant positive correlation, which could imply related environmental exposures or similar metabolic handling within the body. Ni’s neuroprotective functions include supporting enzymatic processes and reducing oxidative stress, which are crucial for maintaining neuronal health and function (32, 33). Adequate Ni levels may help in the synthesis of serotonin and dopamine, neurotransmitters essential for mood regulation (6, 10). One study showed that serum Ni concentration in MDD patients was significantly lower than that of HC, highlighting its potential role in mitigating depressive symptoms (34).

Elevated Manganese levels in serum and urine were significant protective factors against MDD. Mn is crucial for various biological processes, including neurotransmitter synthesis and antioxidant defense mechanisms. Mn acts as a co-factor for enzymes involved in the synthesis of neurotransmitters such as dopamine and serotonin, which play key roles in mood regulation (35). However, excessive Mn exposure can lead to neurotoxicity, known as manganism, which resembles Parkinson’s disease and includes symptoms of severe depression and anxiety (36). A study, for example, found an association between urinary manganese levels and depressive symptoms in adults (22). Therefore, while adequate Mn levels are protective, maintaining a balance is essential to avoid toxicity (37).

Cadmium is a toxic heavy metal that can accumulate in the body, particularly in the kidneys and liver, causing damage to various organs and systems (38). Paradoxically, our logistic regression analysis indicated that higher serum cadmium levels were significantly associated with a reduced odds of MDD. This finding appears to be inconsistent with the well-established neurotoxic effects of cadmium exposure. While one study has highlighted the potential role of cadmium in inducing oxidative stress and modulating antioxidant enzyme activity, which could theoretically exert neuroprotective effects (39), the overall evidence on the relationship between cadmium and mental health is conflicting. High-dose cadmium exposure remains neurotoxic and detrimental to mental health (40, 41). A study has suggested that cadmium exposure may influence depression through metabolic pathways such as inducing oxidative stress, inflammation, and disrupting amino acid metabolism (42). However, the observed inverse association between cadmium levels and MDD risk in our study warrants further investigation to elucidate the underlying mechanisms and rule out potential confounding factors. We cannot make definitive conclusions about the neuroprotective effects of cadmium based on our current findings alone.

Lead, Cesium, and Mercury are EMs with known neurotoxic effects and potential links to depression (5, 43). Previous research indicates that Pb exposure can alter the hypothalamic–pituitary–adrenal axis, affecting glucocorticoid and catecholamine levels, thereby increasing the risk of depression (44). Mercury, a neurotoxic heavy metal, affects the nervous system through various forms and mechanisms. Overwhelming evidence suggests that mercury exposure is detrimental to mental health and increases the risk of depression, with only a few studies showing neutral or contradictory results potentially influenced by factors like dosage, exposure duration, and individual susceptibility (32, 45–48). Cs has been identified as a critical metal in classifying depression, with studies showing that higher urinary cesium levels are associated with cognitive performance decline and a lower prevalence of cognitive impairment (13, 40, 49).

In our study, we found that there was a positive correlation between serum Pb concentration and depression in patients with moderate to severe depression. While no significant differences in Hg concentrations were observed between individuals with MDD and HCs, our analyses revealed that elevated Hg levels were significantly associated with increased severity of depressive symptoms at the median level. However, for Cs, higher serum Cs levels were significantly associated with a lower odds of MDD, suggesting a protective role against depression. Serum Cs and urinary Ni were found to have a significant negative correlation, indicating potential competitive interactions or divergent metabolic pathways influencing their concentrations.

### 4.3 Metabolic insights from serum and urine EM discrepancies

The differences in EM concentrations between serum and urine suggest distinct metabolic processes. Serum EM levels reflect recent exposure and immediate metabolic response, while urine levels indicate excretion and detoxification status. The discrepancy between serum and urine metal levels suggests a complex role for these elements, potentially influenced by their compartmentalization within the body or differing metabolic pathways (31). In urine, certain metals may be rapidly excreted, reflecting recent exposure rather than long-term accumulation and toxicity (50).

Lower serum levels of Cs and Cd in MDD patients might suggest altered absorption or retention, whereas higher Sn levels could indicate increased exposure or impaired clearance. Higher Sn and lower Ni levels in urine among MDD patients suggest differences in renal handling and excretion. Reduced urinary excretion of Ni may indicate decreased intake or enhanced retention, while elevated Sn levels could reflect a compensatory mechanism to remove excess Sn.

These metabolic differences highlight the need for a dual approach in assessing metal exposure. Blood samples provide a snapshot of immediate exposure, while urine samples offer insights into longer-term excretion processes (50). Understanding these pathways is crucial for managing EM exposure and its effects on mental health, particularly in MDD.

### 4.4 Limitations

This study still has several limitations. Firstly, the sample size of this study is relatively small, and the small sample size at each quantile point in the quantile regression may result in findings that lack generalizability. Secondly, as a cross-sectional study, it is difficult to establish causal relationships. Furthermore, the exclusion criteria of this study did not include inflammatory diseases and the use of other medications and supplements. Inflammation may significantly affect the levels of EMs in serum and urine, and certain medications and nutritional supplements may interfere with the absorption, metabolism, or excretion of EMs, thereby affecting their levels. The study did not rigorously collect data on comorbidities during the sample collection phase because of the fragmentation of the medical records. Despite these challenges, we have taken several steps to mitigate this limitation. We collected data on other health-related aspects through various validated scales: SHAPS, GAD-7, PSQI and BISS. Additionally, some other elements may indirectly affect the levels of EMs in the human body; for example, iodine intake can affect thyroid function and thus influence individual metabolism. The study only analyzed the levels of eight EMs in serum and urine, without considering the relationship between other EMs such as iron and magnesium and depression. EM levels in other body parts, such as nails and hair, are also associated with mental disorders such as depression, warranting further investigation through multimodal research to explore these mechanisms. Finally, our study did not control for participants’ diets. Despite all participants residing in the same place with presumed similar dietary habits, individual variations in diet and environmental factors could affect the generalizability of our findings.

## 5 Conclusion

Our findings reveal significant differences in EM concentrations between MDD patients and healthy controls, and also identify correlations among them, showing either synchronous or divergent trends. Elevated serum and urine levels of Sn in MDD patients suggest it as a potential risk factor, whereas higher levels of Cs, Cd, Mn, Ba, and Ni may confer some protection against MDD. Moreover, increased concentrations of Ba, Sn, Pb, Hg, and Mn are linked to more severe depressive symptoms, underscoring their influence on disease severity. These results highlight the need for further research into the specific metabolic mechanisms of EMs in MDD, which could lead to new prevention and treatment strategies.

## Data Availability

The raw data supporting the conclusions of this article will be made available by the authors, without undue reservation.
